# The Roles of DNA Topoisomerase IIβ in Transcription

**DOI:** 10.3390/ijms19071917

**Published:** 2018-06-29

**Authors:** Ram Madabhushi

**Affiliations:** Department of Psychiatry, University of Texas Southwestern Medical Center, Dallas, TX 75390, USA; ram.madabhushi@utsouthwestern.edu; Tel.: +1-214-648-6508

**Keywords:** DNA topoisomerase, topology, DNA double strand breaks, transcription

## Abstract

Type IIA topoisomerases allow DNA double helical strands to pass through each other by generating transient DNA double strand breaks βDSBs), and in so doing, resolve torsional strain that accumulates during transcription, DNA replication, chromosome condensation, chromosome segregation and recombination. Whereas most eukaryotes possess a single type IIA enzyme, vertebrates possess two distinct type IIA topoisomerases, Topo IIα and Topo IIβ. Although the roles of Topo IIα, especially in the context of chromosome condensation and segregation, have been well-studied, the roles of Topo IIβ are only beginning to be illuminated. This review begins with a summary of the initial studies surrounding the discovery and characterization of Topo IIβ and then focuses on the insights gained from more recent studies that have elaborated important functions for Topo IIβ in transcriptional regulation.

## 1. Discovery of DNA Topoisomerase IIβ

Topo IIβ was discovered serendipitously during attempts to purify topoisomerase II activity from amsacrine-resistant cells. At the time, it was recognized that several structurally diverse classes of antitumor drugs markedly increase the formation of Topo II-associated strand breaks in DNA, and cell lines that developed resistance to one class of Topo II-active compounds were also cross-resistant to other Topo II-active, but not Topo I-active, compounds. These and other observations led Drake, Mirabelli, and colleagues to speculate that the resistance to these drugs could arise from changes in either the expression or the activity of Topo II [1]. Indeed, their studies on amsacrine-resistant murine P388 leukemia cells revealed that the levels of the originally identified 170 kDa isoform of Topo II was significantly reduced in these cells. However, they noticed that in addition to this 170 kDa polypeptide, Topo II activity was also detected in a distinct 180 kDa polypeptide that could be distinguished from the 170 kDa isoform in antigenicity, proteolytic cleavage patterns, enzymatic properties, and sensitivity to pharmacological Topo II inhibitors [1,2]. These studies strongly suggested that the two polypeptides represent two distinct isoforms of Topo II. Subsequently, sequencing of partial cDNA clones from a human Raji-HN2 cDNA library revealed two classes of nucleotide sequences that hybridized to unique non-overlapping DNA restriction enzyme fragments in Southern blotting experiments [3]. Similar results were reported from the sequencing of partial cDNA clones from a HeLa carcinoma cDNA library [4]. Together, these studies provided genetic evidence that the two Topo II isoforms could be encoded by two different genes. Whereas the gene encoding human Topo IIα had been mapped to chromosome 17 (17q21-22), further cloning and gene mapping efforts localized the gene encoding human Topo IIβ to chromosome 3 (3p24), and verified this idea [5,6,7]. Accordingly, the original 170 kDa isoform of Topo II was designated as Topo IIα, and the 180 kDa isoform as Topo IIβ [3], and the corresponding genes were named *TOP2A* and *TOP2B*, respectively.

Whereas reduction in Topo IIα levels led to amsacrine resistance in murine P388 leukemia cells as described above [1], several mutations in *TOP2A* were also associated with the development of drug resistance in human cancers. These observations prompted a deeper investigation into the genomic organization of *TOP2A* and *TOP2B* with the notion that such knowledge could help with mutation detection and alternative treatment strategies in patients with drug-resistant cancers [8,9]. However, these studies also provided several important insights into the evolution of the two Topo II isoforms. Comparisons of intron positions and intron-exon organization between *TOP2A* and *TOP2B* revealed a high degree of similarity [8,9], and the amino acid sequences of TOP2A across vertebrates were found to be more similar to each other than to the sequences of TOP2B within the same species. Together, these results suggest that *TOP2A* and *TOP2B* likely arose from the duplication of an ancestral gene [9]. It is thought that eukaryotic Topo II was derived from the fusion of genes analogous to bacterial *gyrA* and *gyrB* that together encode the subunits of bacterial DNA gyrase [10]. It is likely that the gene duplication event that yielded *TOP2A* and *TOP2B* occurred prior to the evolution of vertebrates given that lower eukaryotes, including yeast, flies, and worms, have only one Topo II isoform, whereas vertebrates possess two Topo II isoforms. Interestingly, amino acid sequence alignments also revealed a greater inter-species divergence among TOP2A sequences compared to the divergence between TOP2B sequences, indicating that *TOP2B* genes are under stronger selection pressure than *TOP2A* genes [8].

## 2. Distinctions between Topo IIα and Topo IIβ

The presence of two Type II topoisomerases in vertebrate cells raises the question of whether they are utilized to perform specialized and non-redundant roles. Early studies in synchronously growing cells revealed that Topo IIα levels oscillate during the cell cycle, with the levels increasing during S, G2, and M phases of the cell cycle and decreasing as cells entered either G1 or G0 [11]. In contrast, Topo IIβ levels vary little with cell cycle progression and increase as cells enter quiescence [11]. As cells enter mitosis, Topo IIα becomes tightly chromosome-bound whereas Topo IIβ displays a diffuse cytosolic distribution during metaphase and is visible again post-mitotically following nuclear assembly [12,13]. In fact, unlike the loss of Topo IIα, the loss of Topo IIβ does not affect cell proliferation [14]. Furthermore, Topo IIβ is unable to rescue the mitotic defects in human H69-VP cells that arise from mutations in Topo IIα [15]. These observations suggest that cells preferentially utilize Topo IIα during mitosis, and that Topo IIβ does not adopt these functions in the absence of functional Topo IIα.

In parallel to the assessment of Topo IIα and Topo IIβ dynamics during the cell cycle, assessments of the distribution of the two isoforms across various mammalian tissues also suggest that the two isoforms play distinct biological roles [16,17,18,19,20]. Northern blot analysis of Topo IIα and Topo IIβ expression in mice indicated that the expression of Topo IIα was restricted to a few tissues, notably those characterized by proliferating cells, such as the bone marrow, intestine, and spleen, whereas Topo IIβ expression was detected in most adult tissues [16]. Similarly, in situ hybridization experiments with isoform-specific oligonucleotide probes in the developing rat brain revealed that Topo IIα expression is prominent within the ventricular zones of various brain regions at early embryonic stages and in the external granular layer of the cerebellum [18]. The ventricular zone of the cerebral cortex and the external granular layer of the cerebellum consist of proliferating neural progenitors that divide to produce post-mitotic neurons, which subsequently divide and migrate to their final destinations. In contrast to the selective expression of Topo IIα in these proliferative zones, Topo IIβ mRNA was observed to be distributed throughout the brain [18]. These results were further established from in situ hybridization experiments in fetal human tissues, which again revealed that Topo IIβ is more widely expressed, whereas Topo IIα expression is enriched within the proliferative zones of various tissues [19]. Taken together, the studies on cell cycle dynamics and tissue distribution indicate Topo IIβ is the more ubiquitous Topo II isoform, and that Topo IIα is the more “specialized” isoform that is utilized for challenges that arise during cell cycle progression, such as DNA replication, chromosome condensation, and chromosome segregation.

The mechanistic underpinnings for the utilization of two Type IIA topoisomerases in vertebrates for distinct cellular functions is not understood, although this scenario is not unique to vertebrates. For instance, bacteria contain two type IIA topoisomerases, DNA gyrase and Topoisomerase IV (Topo IV), that perform distinct functions—DNA gyrase is primarily responsible for the removal of positive supercoils ahead of moving DNA/RNA polymerases, whereas the crucial role of Topo IV is to decatenate daughter chromosomes following DNA replication [21]. These distinct cellular roles arise from important structural differences between DNA gyrase and Topo IV. Specifically, the C-terminal domain of the DNA gyrase A subunit confers the enzyme with the unique ability to wrap DNA. The right-handed DNA wrapping by DNA gyrase introduces a crossover between flanking DNA segments and favors intramolecular strand passage reactions over intermolecular events [21]. On the other hand, Topo IV is more effective than DNA gyrase at resolving intermolecular entanglements, such as knots, precatenanes, and catenanes [21].

In contrast to this scenario, biochemical and crystallographic studies indicate that human Topo IIα and Topo IIβ display very similar catalytic and structural properties [1,2,22,23]. Furthermore, although Topo IIβ cannot compensate for the loss of Topo IIα in mammalian cells, both Topo IIα and Topo IIβ are able to complement the loss of Topo II function in conditional *top2* mutants in *Saccharomyces cerevisiae* [24,25]. These observations suggest that the unique cellular roles for Topo IIα and Topo IIβ likely arise from the differential regulation of the two isozymes, and not from differences in core catalytic properties. 

Topo IIα and Topo IIβ sequences are broadly conserved within the ATPase and DNA cleavage and religation domains, whereas the C-terminal and extreme N-terminal regions are the least conserved between the two isoforms [26]. These observations formed the rationale for investigating whether divergence within the C-terminal region (CTR) of Topo II could explain the functional differences between Topo IIα and Topo IIβ. Truncations of the CTR do not affect the catalytic activity of either Topo IIα or Topo IIβ, and similar results have been reported for Topo II from yeast and *Drosophila* [27,28,29,30,31,32]. However, the CTR is necessary and sufficient for the nuclear localization of both Topo IIα and Topo IIβ, and several putative nuclear localization sequences have been identified within this domain [26,31,33]. Elegant “tail-swap” studies, in which the CTRs of Topo IIα and Topo IIβ are exchanged to produce chimeric proteins, revealed that the CTR is responsible for cell cycle-dependent behavior of the two isoforms [33,34]. For instance, all proteins bearing the Topo IIα CTR were able to support cell proliferation and became tightly associated with chromosomes during metaphase, whereas proteins bearing the Topo IIβ CTR could only weakly support proliferation and were not chromosome-bound in metaphase [33]. Further in vitro characterization of these truncated and chimeric Topo IIs showed that although the loss of the CTR did not affect the overall catalytic activity of either Topo IIα or Topo IIβ, truncation of the βCTR markedly increased the binding of Topo IIβ to DNA, whereas truncation of the αCTR had no effect on DNA binding by Topo IIα [35]. Moreover, fusion of βCTR to Topo IIα markedly inhibited the catalytic activity of Topo IIα, whereas the fusion of the αCTR to Topo IIβ mildly stimulated its activity [34]. Together, these observations suggest that the distinct cell cycle-dependent properties of Topo IIα and Topo IIβ are determined by their divergent CTRs, and that the CTR of Topo IIβ could be a negative regulator of Topo IIβ.

The precise mechanisms by which the CTR influences the activity of Topo II isoforms are still poorly understood. The CTR is the site of many posttranslational modifications, including phosphorylation and SUMOylation. Recent studies suggest that the modification of Topo IIα CTD through SUMOylation could be crucial for its roles in mitosis [36]. On the other hand, based on sequence analysis, thirty potential protein kinase C phosphorylation sites and about forty potential casein kinase II phosphorylation sites were identified for Topo II [26]. Both Topo IIα and Topo IIβ are phosphorylated in a cell cycle-dependent manner, and several cell cycle-dependent phosphorylation sites have been identified in Topo IIα [26]. Increased phosphorylation of Topo IIβ has been reported in human leukemia HL-60 cells that were induced to differentiate using all-trans retinoic acid, and in doxorubicin-resistant HL-60 cells, and increased Topo IIβ phosphorylation was shown to correlate with increased protein stability during retinoic acid-induced differentiation [37,38]. Similarly, increased Topo IIβ levels were also reported in an acute promyelocytic leukemia (APL) line that was resistant to treatment with retinoic acid, and these increased levels were attributed to protein kinase C-δ (PKCδ)-mediated phosphorylation of Topo IIβ [39,40]. However, the role of the CTR and the effect of posttranslational modifications on the activity and function of Topo IIβ need further characterization.

## 3. Genetic Studies Reveal a Role for Topo IIβ in Neural Development

While investigations into the cell cycle dynamics and tissue distribution of Topo IIα and Topo IIβ proved extremely important for clarifying the role of Topo IIα, they were less successful in identifying precise cellular functions for Topo IIβ. In an attempt to determine the function of Topo IIβ in vivo, Yang, Wang and colleagues disrupted the murine *Top2b* gene [41]. Although heterozygous *Top2b^+/−^* mice were indistinguishable from their wild-type littermates, homozygous *Top2b^−/−^* pups succumbed to respiratory failure shortly after birth [41]. Detailed examination of *Top2b^−/−^* embryos at various stages revealed marked neurological defects, including the failure of motor neurons to innervate skeletal muscles and of sensory neurons to enter the spinal cord [41]. Meanwhile, further characterization of Topo II isoform expression patterns during cerebellar development that extended upon early tissue distribution studies uncovered a sharp transition from Topo IIα to Topo IIβ expression as cells exited the cell cycle and began their differentiation into either granule or Purkinje cells [42]. Together, these observations suggested that Topo IIβ is essential for neural development. 

This hypothesis was tested with the generation of a conditional mouse model that allowed for the selective ablation of *Top2b* in the brain [43]. These brain-specific *Top2b^−/−^* embryos (*bTop2b* KO) also displayed the perinatal death from respiratory failure that was observed in mice lacking *Top2b* in all tissues, although their body size and appearance were similar to *Top2b^+/+^* embryos (embryos lacking *Top2b* in all tissues are significantly smaller than their *Top2b^+/+^* counterparts) [43]. Furthermore, an examination of corticogenesis in *bTop2b* KO mice revealed substantial defects in cortical lamination [43]. During neuronal development in the cerebral cortex, cortical layering proceeds in an “inside-out” fashion (designated layers I-VI beginning from outer brain surface). Neural progenitors divide and produce neurons within the ventricular and sub-ventricular zones, and the newborn neurons organize themselves into six layers by following a systematic migration pattern in which later born neurons migrate past and populate cell layers above those formed by early born neurons [44]. In *bTop2b* KO embryos, neurons born at later stages failed to migrate to their appropriate destinations, and *bTop2b* KO embryos were characterized by a thinner cortex, layering defects in the hippocampus, and defective development of the olfactory bulb [43]. Furthermore, Topo IIβ was shown to be essential for neurite outgrowth in cultured rat cerebellar and cortical neurons, differentiating rat PC12 cells, primary mouse ventral mesencephalic neurons, and differentiated human mesenchymal stem cells [45,46,47,48,49]. Mutations in Topo IIβ were also shown to disrupt proper targeting of retinal ganglion cell axons and proper wiring of the visual system in zebrafish [50]. Thus, the results from genetic studies in vivo suggest that Topo IIβ activity is crucial for proper development and function of newly formed post-mitotic neurons. In a recent case report, whole genome sequencing identified a novel *de novo* mutation in *TOP2B* that was associated with developmental delay, intellectual disability, hypotonia, progressive microcephaly, and autistic features [51], suggesting that Topo IIβ could also be essential for proper neuronal development in humans and that mutations in *TOP2B* could lead to neurodevelopmental disorders.

## 4. Topo IIβ and Transcriptional Regulation of Developmental Genes in Neurons

DNA unwinding and tracking of RNAPII introduces superhelical tension in the DNA that manifests in the form of positive supercoils ahead of RNAPII and negative supercoils behind it [52,53]. Both topoisomerase I (Topo I) and Topo II in eukaryotes are capable of resolving both positive and negative supercoils, and their collaborative activities maintain DNA topology in a state that is competent for various template-directed processes, including transcription and DNA replication. Despite these similarities, it was shown that Topo II is more efficient at relaxing supercoils that accumulate in the context of chromatin [54,55], and the association of Topo IIα with RNAPII was shown to be required for transcription on chromatin templates in vitro [56]. Within this context, attempts to explain how the loss of Topo IIβ could lead to neurological phenotypes has been centered on the hypothesis that Topo IIβ activity is crucial for the expression of developmentally regulated genes in postmitotic neurons. Based on similarities between the abnormal cerebral stratification phenotypes of *Top2b^−/−^* embryos and mutants that are defective in reelin signaling, the expression of reelin was examined in reelin-secreting Cajal-Retzius cells in the neocortex of *Top2b^−/−^* embryos and its levels were found be reduced compared to reelin expression in *Top2b^+/+^* embryos [43]. Likewise, the inhibition of Topo IIβ in differentiating cerebellar neurons was shown to attenuate the transcription induction of amphyphysin I, which facilitates clathrin-mediated endocytosis of synaptic vesicles [57]. These initial “single-gene” studies formed the basis for more detailed investigations into the roles of Topo IIβ in transcription in the nervous system [58,59,60,61]. Microarray analysis of gene expression profiles from *Top2b^−/−^* mouse brains at various embryonic stages showed that the expression of about 30% of the developmentally regulated genes was affected, including genes that encoded for axon guidance, ion channels, and synaptic transmission [58]. Chromatin immunoprecipitation (ChIP) experiments at later embryonic stages revealed preferential binding of Topo IIβ to the 5′ regions of a number of Topo IIβ-sensitive genes, and supported a model in which Topo IIβ activity at the 5′ regions of developmentally regulated neuronal genes, especially in the promoter regions, could regulate their transcription [58].

Meanwhile, transcriptional induction during differentiation and susceptibility to the Topo II inhibitor, ICRF-193, was utilized to identify developmentally induced Topo IIβ-regulated genes in rat cerebellar neurons (Topo IIα is not expressed in these neurons at the time of ICRF-193 addition) [59]. In parallel, genomic sites occupied by active Topo IIβ were mapped by using etoposide to first trap Topo IIβ-DNA covalent cleavage complexes, followed by DNA fragmentation, immunoprecipitation of Topo IIβ-DNA complexes, and hybridization of the associated DNA to tiling arrays [59]. These studies uncovered that Topo IIβ-regulated developmental genes are enriched for long genes that preferentially reside adjacent to long AT-rich intergenic regions, and are typically membrane proteins that encode for ion channels and receptors [59]. Notably, the specific requirement of Topo IIβ (and Topo I) for the expression of long genes has subsequently been reported for cortical primary neurons and during the development of cerebellar neurons [61,62]. Although only a limited number of Topo IIβ sites were probed in the tiling array, superimposition of the gene expression and active Topo IIβ binding sites showed an enrichment of Topo IIβ binding adjacent to Topo IIβ-sensitive developmental genes and within their adjacent long AT-rich intergenic regions [59]. From these results, it was suggested that a higher-order chromatin structure within these AT-rich intergenic regions would likely curtail gene expression and that Topo IIβ activity at the appropriate time during would override this repression [59].

In a further effort to understand whether Topo IIβ binding patterns during neural development could explain the phenotypes of *Top2b^−/−^* animals, *Top2b^−/−^* mouse embryonic stem cells (ESCs) were obtained from the progeny of *Top2b^+/−^* mice, and gene expression changes were assessed as the ESCs differentiated into neural progenitors (NPCs) and postmitotic glutamatergic neurons [60]. Whereas no significant changes in gene expression were detected in ESCs and NPCs lacking Topo IIβ, genes that played a role in neurogenesis were downregulated and neurons showed signs of degeneration, ultimately undergoing apoptotic death [60]. In this study, Topo IIβ binding patterns were examined on a wider scale using ChIP with Topo IIβ-specific antibodies, followed by hybridization to custom tiling arrays spanning well-annotated promoters in the mouse genome, large multigene loci, and the complete chromosome 19 [60]. In contrast to the binding of Topo IIβ to AT-rich intergenic regions that was reported for cerebellar neurons [59], and more similar to limited ChIP-based studies in embryonic brains [58], Topo IIβ binding was shown to be preferentially enriched within promoters that contain chromatin marks of actively transcribed regions, such as dimethylated lysine 4 on histone H3 (H3K4me2), and also recruit RNA polymerase II (RNAPII) [60]. Furthermore, a substantial number of Topo IIβ-bound genes in wild-type neurons were downregulated in *Top2b^−/−^* neurons, indicating that Topo IIβ activity facilitates the induction of these genes [60]. 

The reasons behind some of the differences between reported Topo IIβ binding profiles in these studies could arise from the fact that only a subset of genome-wide Topo IIβ binding sites were probed in each study, and that designs of the tiling arrays were to some extent informed by the nature of observed gene expression changes. Recent next-generation sequencing-based approaches have overcome the limitations of using custom tiling arrays, and have provided a more comprehensive understanding of Topo IIβ binding patterns and activity. The utilization of chromatin immunoprecipitation followed by next-generation sequencing (ChIP-seq) in various cell types, including primary mouse cortical neurons, mouse cerebellar granule neurons, primary mouse liver cells, B-cells from mouse spleen, and human MCF-7 cells, has shown that while Topo IIβ binding is increased within the promoters of actively transcribed genes, it is more broadly distributed across regions of open chromatin [62,63,64,65,66]. Analysis of Topo IIβ occupancy in primary mouse cortical neurons under basal conditions and following neuronal stimulation revealed increased Topo IIβ binding within sequences upstream of actively transcribed genes, including promoters and enhancers, and Topo IIβ binding patterns overlapped with those of transcription factors that regulate neuronal activity-dependent gene expression, including CREB, SRF, and CBP, as well as the chromatin architectural protein, CTCF [63]. In cerebellar granule neurons, Topo IIβ was shown to bind upstream sequences of long genes and to collaborate with the chromatin remodeler, CHD7, to facilitate the expression of these long genes [62]. Similar to the results from mouse cortical neurons, analysis of Topo IIβ ChIP-seq profiles in primary mouse liver cells revealed an enrichment of Topo IIβ binding within the promoters of highly expressed and actively transcribed genes, with liver-specific transcription factors, and with binding sites for CTCF and cohesin [64]. The enrichment of Topo IIβ at the promoters of highly expressed genes is also consistent with studies that have assessed the relationships between supercoiling, transcription, and topoisomerases, and have found that the loss of Topo II activity affects supercoiling and transcription of either the most highly expressed genes or extremely long genes [61,62,67,68,69]. In human MCF-7 cells, 50% of Topo IIβ peaks were detected either within a gene or within 5 kb of a transcription start site. In the absence of stimulation with estradiol, a substantial portion of Topo IIβ was found to be associated with transcription start sites, and additionally 4.3% and 3% of Topo IIβ peaks were detected at CpG islands, and at sites occupied by H3K4me1, respectively [65]. Transcription factor motif analysis revealed binding sites for CTCF, SP1, KLF4, and TFAP2 to be enriched at Topo IIβ-bound loci [65].

Taken together, two major conclusions can be drawn from both the so-called single gene and larger studies: (1) Topo IIβ activity is especially crucial for the late stages of neuronal differentiation; (2) within this developmental period, the loss of Topo IIβ only affects the expression of a selective subset of neuronal genes. Thus, the model that emerges from these studies is that an increase in Topo IIβ levels in newly formed postmitotic neurons is associated with the targeting of Topo IIβ to selective genomic regions, that in turn, facilitates the expression of gene products that are essential for neuronal differentiation and survival.

## 5. Topo IIβ and Transcriptional Regulation through the Formation of Stimulus-Induced DSBs

Whereas both the levels and activity of Topo IIβ in cerebellar neurons peak during the differentiation of Purkinje and granule cells, and decline thereafter, approximately half the peak expression level of Topo IIβ is maintained into adulthood [57]. In fact, as mentioned above, Topo IIβ expression can be detected in most adult tissues [16]. However, the precise post-developmental functions of Topo IIβ are not properly understood. 

In this regard, Ju, Rosenfeld, and colleagues reported a surprising requirement for Topo IIβ-mediated DNA cleavage in the expression of oestrogen receptor-α (ERα) target genes [70]. Using ChIP to analyze the promoter of the estrogen-responsive gene, *pS2,* in MCF-7 human breast cancer cells, they found that stimulation of these cells with estradiol caused the rapid recruitment of ERα and Topo IIβ at the *pS2* promoter [70]. Unexpectedly however, they observed that Topo IIβ activity results in the formation of transient DNA double strand breaks (DSBs) within the promoter that could be detected by end-labeling experiments, and that this results in the subsequent recruitment of DSB repair factors, including Ku70, Ku80, DNA-PK, and PARP1 [70]. Both DSB formation and *pS2* induction in the presence of estradiol were blocked when the activity of Topo IIβ was inhibited [70]. These results suggested that stimulation with estradiol caused Topo IIβ to induce transient DSBs that are not religated by the enzyme itself but are instead repaired using classical DSB repair pathways, such as nonhomologous end joining (NHEJ). They further reported that promoters of other stimulus-responsive genes, including genes that are targets of androgen receptor (AR), retinoic-acid receptor (RAR), and thyroid hormone receptor (T3R) also incur stimulus-induced DSBs within their promoters [70]. Similar Topo IIβ-mediated DSBs have also been reported to form in the promoters of genes stimulated by androgens, insulin, glucocorticoids, retinoic acid, and serum, indicating that Topo IIβ-mediated DSBs are formed in response to diverse physiological stimuli [39,71,72,73,74]. In each case, Topo IIβ activity was also essential for the stimulus-dependent induction of genes that incur DSBs within their promoters. Together, these studies uncovered an important physiological role for Topo IIβ, and suggest that although Topo IIβ is dispensable for cell proliferation, its activity is crucial for the transcriptional induction of stimulus-responsive genes in proliferating cells.

Meanwhile, originating from an unrelated line of reasoning, investigations in postmitotic neurons arrived at similar conclusions. Several studies noted that the stimulation of neurons using various paradigms led to the formation of DNA DSBs [63,75,76]. For instance, DSB formation was detected following brief incubation of cultured primary neurons with *N*-methyl-d-aspartate (NMDA), which mimics the actions of the neurotransmitter, glutamate, upon depolarization of cultured primary neurons with potassium chloride, in the dentate gyrus following exploratory behavior in mice, in the primary visual cortex after visual stimulation in anesthetized mice, following optogenetic stimulation of the striatum in awake, behaving mice, and in the hippocampus following exposure of mice to an associative learning task, etc. [63,75,76].

A major signaling event in the cellular response to the formation of DNA DSBs involves the phosphorylation of the histone variant, H2AX (termed γH2AX), in the vicinity of DSB sites. This feature was exploited to map the genome-wide locations of stimulus-induced DSBs in neurons [63]. ChIP-seq with antibodies against γH2AX and PCR-based assays suggested that stimulus-induced DSBs form within only a few genomic loci (21 loci were identified when cultured primary neurons were stimulated with NMDA), and these loci were enriched for the promoters of the so-called early response genes (ERGs), such as *Fos*, *Npas4*, and *Egr1*, which are rapidly induced in response to neuronal activity [63]. A substantial number of ERGs encode for transcription factors, which in turn, promote the induction of other neuronal activity-regulated genes, such as *Bdnf*, *Cpg15*, and *Rgs2*, that ultimately mediate experience-driven changes to synapses, and this neuronal activity-dependent transcription program is crucial for the development of lasting adaptations in response to environmental cues, including the formation of long-lasting memories [77,78]. The link between DSB formation within ERG promoters and their transcriptional induction in response to neuronal activity also proved to be dependent on Topo IIβ–ChIP-qPCR and ChIP-seq experiments revealed that Topo IIβ is bound to ERG promoters even under basal conditions and that its binding is stimulated in response to neuronal activity, and knockdown of *Top2b* expression attenuates both stimulus-induced DSB formation and ERG induction [63]. Importantly, the effects of *Top2b* knockdown on ERG expression could be rescued by targeting DSB formation within ERG promoters using CRISPR-Cas9 [63]. Similarly, treatment of neurons with etoposide to trap Topo IIβ cleavage complexes was sufficient to induce modest ERG expression even in the absence of a stimulus [63]. These results suggest that Topo IIβ-mediated DNA cleavage is the key activity that controls transcription induction of ERGs in neurons.

## 6. Mechanisms Underlying Transcriptional Regulation by Topo IIβ

The studies describing the roles of Topo IIβ in the control of developmentally regulated and stimulus-responsive genes raise the question of how Topo IIβ activity leads to transcriptional induction. An obvious mechanism, especially for developmentally regulated genes, could be that Topo IIβ promotes transcriptional elongation through the relaxation of transcription-generated supercoils. However, as mentioned above, the expression of only a small subset of genes is affected by the loss of Topo IIβ, indicating that Topo IIβ is not required for the relaxation of transcription-generated supercoils for a majority of genes. In support, recent studies have suggested that Topo I activity is largely responsible for the resolution of transcription-generated supercoiling, whereas Topo II activity is only required for the most highly expressed genes [67,68]. However, it is unclear whether Topo IIβ-sensitive genes are the most highly expressed genes. An alternative explanation is that Topo IIβ activity facilitates the creation of a chromatin environment that is permissive for transcription initiation. For instance, Topo IIβ, but not Topo IIα, was shown to co-purify with ATP-dependent chromatin assembly factor (ACF), a chromatin remodeler that regulates nucleosome spacing [79,80]. Similarly, it was proposed that Topo IIβ activity is required to relieve a repressive chromatin environment that precludes the expression of developmentally regulated genes juxtaposed to long AT-rich intergenic regions [59]. However, the precise interactions and activities of Topo IIβ that lead to the induction of developmentally regulated genes need further clarification.

**Figure 1:**
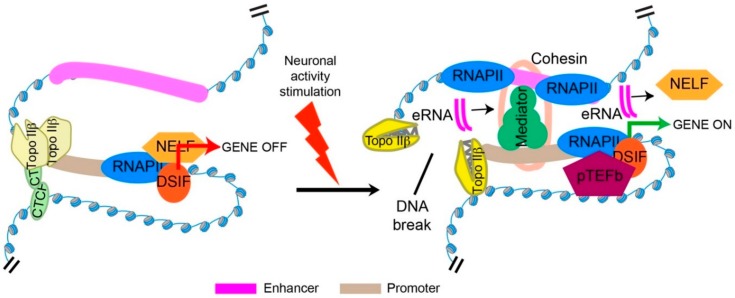
Model depicting the control of gene transcription through Topo IIβ-mediated DNA DSBs. (**Left**) Under basal conditions, RNAPII is held in a paused state through the actions of DSIF and NELF, and enhancer-promoter interactions are precluded through the imposition of a topological constraint by the architectural protein, CTCF. (**Right**) Upon activity stimulation, Topo IIβ-mediated DNA breaks override the CTCF-enforced constraint, and allow for enhancer-promoter interaction. In neurons, this interaction allows for the synthesis of enhancer RNAs (eRNAs) at enhancers, which in turn, mediate the release of NELF. The actions of pTEFb then allow for the escape of RNAPII from the promoter and trigger gene induction. eRNAs have also been shown to stimulate gene expression by stabilizing enhancer-promoter interactions through their interaction with the cohesin and/or mediator complexes, and by modulating chromatin structure at promoters. Adapted with permission from [78].

Likewise, many aspects of the relationship between stimulus-induced DSBs and transcriptional induction also remain unexplored. It is notable that Topo IIβ is already bound to the promoters of ERGs under basal conditions, and the ability of promoter-bound Topo IIβ to become trapped into cleavage complexes upon treatment with etoposide suggests that Topo IIβ is catalytically active at these promoters under basal conditions [63]. These results suggest that the transient DSBs generated by Topo IIβ under basal conditions is not sufficient to trigger transcriptional induction, and that a lasting DSB generated in response to a stimulus could be necessary. In support of this idea, inhibition of NHEJ in cultured primary neurons allowed ERG expression to persist for longer periods of time [63]. These results also suggest that Topo IIβ activity is modulated in a stimulus-dependent manner to generate a lasting DSB. However, precisely how Topo IIβ activity is modulated in a stimulus-dependent manner and how DNA cleavage in this manner facilitates transcriptional induction are understood. In the case of estradiol-induced DSBs within the *pS2* promoter, it was reported that transient DSBs generated by Topo IIβ cause the activation of PARP1 and that PARP1 enzymatic activity mediates a nucleosome-specific exchange of the repressive linker histone H1 for high-mobility group B (HMGB) proteins, which is thought to promote transcriptional activation [70]. As with the regulation of developmentally regulated genes, these results again suggest that Topo IIβ-mediated DSBs could help create a favorable chromatin environment for transcription initiation of stimulus-induced genes. However, it is unclear whether this scenario is applicable to other systems, such as during the induction of ERGs in postmitotic neurons. Two recent developments have provided crucial insights into the regulatory framework that governs the rapid induction of neuronal ERGs. First, the application of next-generation sequencing methods has revealed that unlike what was reported for the *pS2* promoter, neuronal ERGs already possess a chromatin environment that is favorable for transcription under basal conditions, and RNAPII already initiates transcription but resides at promoter-proximal regions in a paused configuration [78,81]. These results suggest that ERG expression is largely controlled at the level of RNAPII pause-release and not at the level of RNAPII recruitment. Second, it has been shown that the release of paused RNAPII requires stimulus-dependent coupling between distally located gene enhancers and the promoters of ERGs. Together, these observations imply a model in which impediments to enhancer-promoter coupling prevent the expression of ERGs under basal conditions, and mechanisms that facilitate enhancer-promoter coupling in response to neuronal stimulation constitute the molecular trigger for the rapid induction of ERGs [78] (Figure 1).

Interestingly, in a recent study that described the formation of stimulus-induced DSBs within the promoters of serum-responsive ERGs, including *FOS* and *EGR1*, inhibition of Topo IIβ was shown to markedly augment promoter-proximal pausing at serum-inducible ERGs, suggesting that Topo IIβ-mediated DSBs could promote RNAPII pause-release [74]. Furthermore, an analysis of sequence properties at genome-wide Topo IIβ binding sites in neurons indicated that the motif for the architectural protein, CTCF, was by the far the most significantly enriched [63]. CTCF was shown to physically interact with Topo IIβ and CTCF binding was enriched at sites that incurred stimulus-induced DSBs in neurons [63]. Based on the roles of CTCF in regulating promoter-enhancer interactions through chromatin looping, it was proposed that CTCF activity at ERG promoters topologically curtails enhancer-promoter interactions under basal conditions and that Topo IIβ-mediated DSBs would override this topological barrier in response to neuronal stimulation by favoring conformational changes that stabilize enhancer-promoter interactions [63,78] (Figure 1). Such a model also explains how Topo IIβ actions could facilitate RNAPII pause-release.

The enrichment of CTCF motifs at Topo IIβ binding sites and the co-occupancy of Topo IIβ and CTCF at the same genomic locations has also been reported from analysis of relevant ChIP-seq datasets in primary mouse liver cells, human MCF-7 breast cancer cells, and primary B cells from mouse spleen [64,65,66]. Detailed analysis of genome-wide Topo IIβ, CTCF, and cohesin binding patterns in primary mouse liver cells revealed that approximately half of all CTCF/cohesin-bound regions were also occupied by Topo IIβ, and these Topo IIβ/CTCF/cohesin bound sites were more likely to be bound by CTCF across multiple tissues and were also evolutionarily more conserved than sites occupied by two of the three components [64]. Furthermore, by reanalyzing available datasets on supercoiling changes under various conditions, it was proposed that Topo IIβ activity could modulate DNA supercoiling at CTCF binding sites [64]. Extending upon these observations, sites of active Topo IIβ in primary B cells were identified by first using etoposide to trap Topo IIβ in cleavage complexes, and then utilizing an assay called END-seq that involves the ligation of biotinylated hairpin adaptors to DNA ends [66]. These studies again revealed that active Topo IIβ is co-localized at sites that are co-occupied by CTCF and cohesin, and suggested that Topo IIβ activity is primarily localized to the anchors of chromatin loops [66]. Interestingly, active Topo IIβ could be trapped even in the absence of transcription. Nevertheless, an enrichment of enhancer-promoter loops was observed at sites of active Topo IIβ [66]. These observations have important implications. They suggest that chromatin organization could be an important source of topological stress and reveal an important physiological role for Topo IIβ in the maintenance of chromatin architecture through the resolution of torsional stress that accumulates during chromatin looping [66]. Additionally, they indicate that although Topo IIβ activity can be detected independent of transcription, the role of Topo IIβ in dissipating torsional stress within chromatin loops could help stabilize enhancer-promoter interactions and facilitate gene expression [66]. Whether this activity could explain the observed effects of Topo IIβ on the expression of developmentally regulated genes is not presently known.

## 7. Conclusions and Future Perspectives

Whereas the initial studies clarified that of the two Topo II isoforms, Topo IIα is selectively utilized and important for chromosome condensation and segregation, more recent studies have revealed specialized roles for Topo IIβ in the maintenance of chromatin architecture, and in transcriptional control of certain developmentally regulated genes, and genes that are induced in response to various external stimuli. However, why vertebrates have evolved two distinct Type IIA topoisomerases for these distinct cellular functions still remains an intriguing question. Studies aimed at further dissecting cellular mechanisms that confer isoform-specific regulation are likely to provide important insights into this matter.

**Figure 2:**
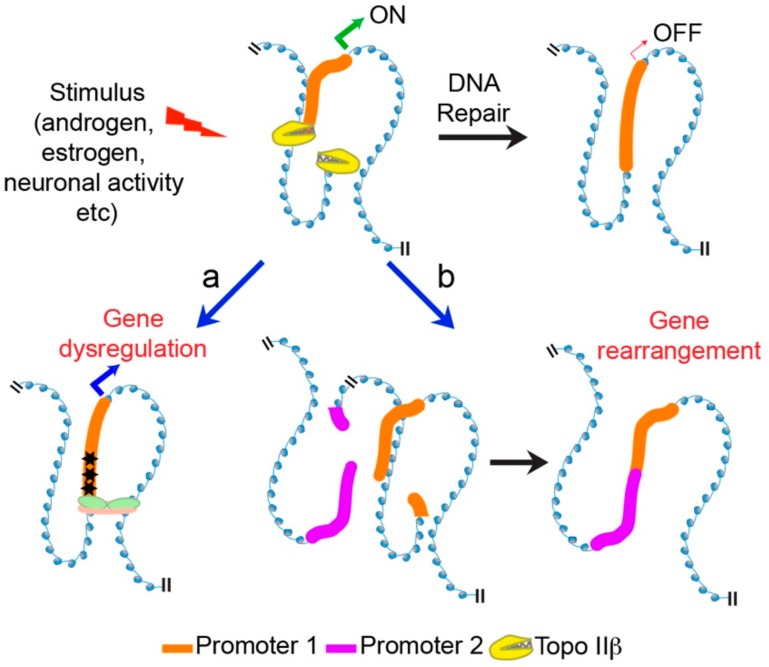
Defective repair of Topo IIβ-mediated DNA DSBs and disease. (Black arrow) The generation and accurate repair of Topo IIβ-mediated DSBs controls activity-induced gene expression in a number of systems. (Blue arrows) Two scenarios by which defective repair of stimulus-induced DSBs could lead to disease are depicted: (**a**) inaccurate repair leads to the accumulation of mutations (black stars) within the promoters of stimulus-responsive genes, and dysregulates their expression. For instance, disruption of ERG induction in neurons could reduce cognitive performance. (**b**) Aberrant recombinogenic repair of activity-induced DSBs could lead to gene arrangements. For instance, more than 50% of prostate cancer patients harbor recurrent gene fusions.

The involvement of Topo IIβ in the maintenance of chromatin architecture and transcription also has important pathophysiological implications. On the one hand, alterations in Topo IIβ levels could affect the transcription of selective genes that govern neurite outgrowth and axonogenesis, and contribute to various neurological disorders [47,82]. On the other hand, the formation of DSBs in the promoters of stimulus-responsive genes during transcription induction suggests that changes in the ability to repair these stimulus-induced DSBs could have detrimental consequences (Figure 2). For instance, failure to accurately repair Topo IIβ-generated DSBs have been linked to genomic rearrangements in prostate cancer, as well as to the development of therapy-related leukemias [66,71,83]. Similarly, failure to accurately repair stimulus-induced DSBs in postmitotic neurons could lead to the accumulation of mutations that might diminish the ability to induce transcription of relevant genes during future rounds of stimulation, and could contribute to the deterioration of neuronal function (Figure 2). The enzyme, tyrosyl-DNA phosphodiesterase 2 (TDP2) mediates the error-free repair of Topo II-generated DSBs [84]. Recently, mutations in *TDP2* were identified in patients that showed characteristic neurological abnormalities, including seizures, cognitive deficits, and ataxia [85]. Whether these defects arise from the inaccurate repair of stimulus-induced DSBs remains to be tested. On the other hand, the identification of mutations in *TOP2B* that are associated with developmental delay and intellectual disability [51] suggest that both the formation and accurate repair of Topo IIβ-generated DSBs are essential for proper function of the nervous system.
